# Genomic epidemiology and plasmid characterization of antimicrobial resistance and virulence in cattle *Escherichia coli* from China

**DOI:** 10.1128/spectrum.03256-25

**Published:** 2025-12-04

**Authors:** Xiang-Yu Wang, Tong Ye, Jian-Gang Ma, Hong-Bo Ni, Li-Gang Xue, Quan Zhao, Li Guo, Xiao-Xuan Zhang

**Affiliations:** 1 Animal Science and Technology College, Jilin Agricultural Science and Technology University 381876, Jilin, Jilin Province, People's Republic of China; 2 College of Veterinary Medicine, Qingdao Agricultural University 98431, Qingdao, Shandong Province, People's Republic of China; 3 College of Veterinary Medicine, Yang Zhou University, Yang Zhou, Jiangsu Province, People's Republic of China; 4 College of Veterinary Medicine, Jilin Agricultural University 85112, Changchun, Jilin Province, People's Republic of China; 5 Xianghu Lab, Hangzhou, Zhejiang Province, People's Republic of China; Earlham Institute, Norwich, United Kingdom

**Keywords:** AMR, *Escherichia coli*, serotype, ST, phylogroups, WGS

## Abstract

Antimicrobial resistance (AMR) in *Escherichia coli* from livestock poses a growing public health threat, yet genomic data on cattle-derived strains in China remain limited. This study investigated AMR, virulence, and plasmid profiles of *E. coli* from diarrheic cattle in four provinces: Anhui (AH), Ningxia (NX), Shandong, and Shanxi (SX). Ninety-one isolates were characterized using antimicrobial susceptibility testing and whole-genome sequencing. Resistance to ampicillin (49.5%), cefotaxime (37.4%), and tetracycline (36.3%) was common, with NX showing the highest resistance rate. Twenty-one multidrug-resistant strains were identified, mainly from NX and SX. Genomic analysis revealed 53 distinct antibiotic resistance genes (ARGs), predominantly *mdf*(*A*), *aph*(6)-*Id*, and *tet*(A), with the highest burdens in NX and SX. Among 196 virulence genes, adherence (*fim* and *csg*) and secretion systems (*espX*, *espR*) predominated, with AH showing the greatest diversity. Plasmid profiling detected 37 replicon types, with incompatibility FIB (IncFIB) being the most abundant. A strong correlation between plasmids and ARGs was found (*r* = 0.626, *P *< 0.001), with key ARGs [*bla*_CTX-M-55_, *tet*(A)] located on IncI1 and IncX1 plasmids, while most virulence genes were chromosomal. Molecular typing identified 45 sequence types (STs) and 59 serotypes, with ST29 (O26:H11) unique to AH and ST1011 (O86:H51) to NX. Phylogenetic analysis revealed clustering by phylogroup, with shared STs and serotypes across regions, indicating clonal and geographic dissemination. These findings underscore the genomic diversity and dissemination risk of AMR *E. coli* in Chinese cattle, highlighting the need for region-specific surveillance.

**IMPORTANCE:**

The growing threat of antimicrobial resistance (AMR) in *Escherichia coli* from livestock raises serious concerns for both animal and public health, especially under the One Health framework. Genomic information on cattle-derived *E. coli* in multi-regions of China has been limited, hindering our understanding of regional AMR patterns. This study addresses that gap by analyzing isolates from diarrheic cattle across four provinces, uncovering clear geographic variation in resistance profiles, virulence traits, and plasmid content. The identification of clinically relevant resistance genes such as *bla*_CTX-M-55_ and *tet*(A) on plasmids indicates a high potential for horizontal gene transfer. The strong association between plasmid types and resistance gene burden highlights key targets for surveillance. These findings offer valuable insights into the molecular epidemiology of bovine *E. coli* and support more effective, region-specific strategies to monitor and control the spread of AMR in livestock.

## INTRODUCTION

*Escherichia coli,* typically a harmless gut commensal, can become a pathogenic and drug-resistant organism, especially in contexts of immunosuppression and excessive antimicrobial use in livestock production (1, 2). Antimicrobial resistance (AMR) in *E. coli* has emerged as a major global public health concern, particularly due to its capacity to acquire and disseminate resistance genes (3, 4). Livestock is increasingly recognized as important sources of multidrug-resistant (MDR) *E. coli*, contributing significantly to environmental and zoonotic transmission (5, 6). Cattle, as key livestock animals, harbor increasingly alarming levels of antimicrobial-resistant *E. coli*, with studies showing a direct link between antimicrobial usage intensity and the abundance of resistance genes in these populations (7, 8). Although some studies have examined the characteristics of bovine *E. coli* resistance in certain regions of China, comprehensive genomic investigations across multiple provinces remain scarce (9, 10).

Horizontal gene transfer (HGT) plays a crucial role in the rapid dissemination of antibiotical resistance genes (ARGs) among bacterial populations, with plasmids serving as key mobile genetic elements that facilitate the transfer of these resistance determinants across diverse strains and species (11). Previous studies have demonstrated that plasmids belonging to the IncL/M, IncI, IncX, and IncHI incompatibility groups serve as key vectors for the dissemination of critical ARGs, including *bla*_NDM_, *mcr*, and *tet*(X3/X4) variants (12, 13). Although the majority of virulence factor genes (VFGs) are chromosomally encoded, some plasmids have also been shown to carry virulence determinants alongside ARGs, thereby enhancing bacterial adaptability and increasing the potential zoonotic risk (14, 15). Understanding plasmid-mediated gene transfer is vital for tackling AMR and preventing the emergence of more virulent, drug-resistant pathogens.

*E. coli* is commonly classified based on sequence types (STs), serotypes, and phylogroups, each providing distinct insights into the population structure (16, 17). Among these, certain types (ST131 and ST29) are notable for their association with increased virulence, AMR, and zoonotic potential (18, 19). Similarly, specific serotypes (O26:H11) have been linked to pathogenicity and epidemiological significance (20). Phylogroup classification provides an additional framework for understanding the population structure of *E. coli* and is often associated with the sources of isolation (21). Additionally, phylogenomic and comparative genomic analyses have become powerful tools to decipher clonal expansions, evolutionary relationships, and transmission patterns of strains across different hosts and geographic regions (22). These approaches provide critical insights into the evolutionary trajectories, transmission dynamics, and epidemiological relationships of pathogenic *E. coli* strains across hosts and regions.

Here, we performed antimicrobial susceptibility testing, whole-genome sequencing, and extensive genomic analysis of *E. coli* from diarrheic cattle across four Chinese provinces to systematically assess ARGs, VFGs, plasmid replicons, molecular types, and phylogenetic relationships, aiming to uncover regional transmission patterns and inform targeted AMR surveillance in livestock systems.

## RESULTS

### Isolation and antimicrobial resistance of *E. coli*

**Fig 1:**
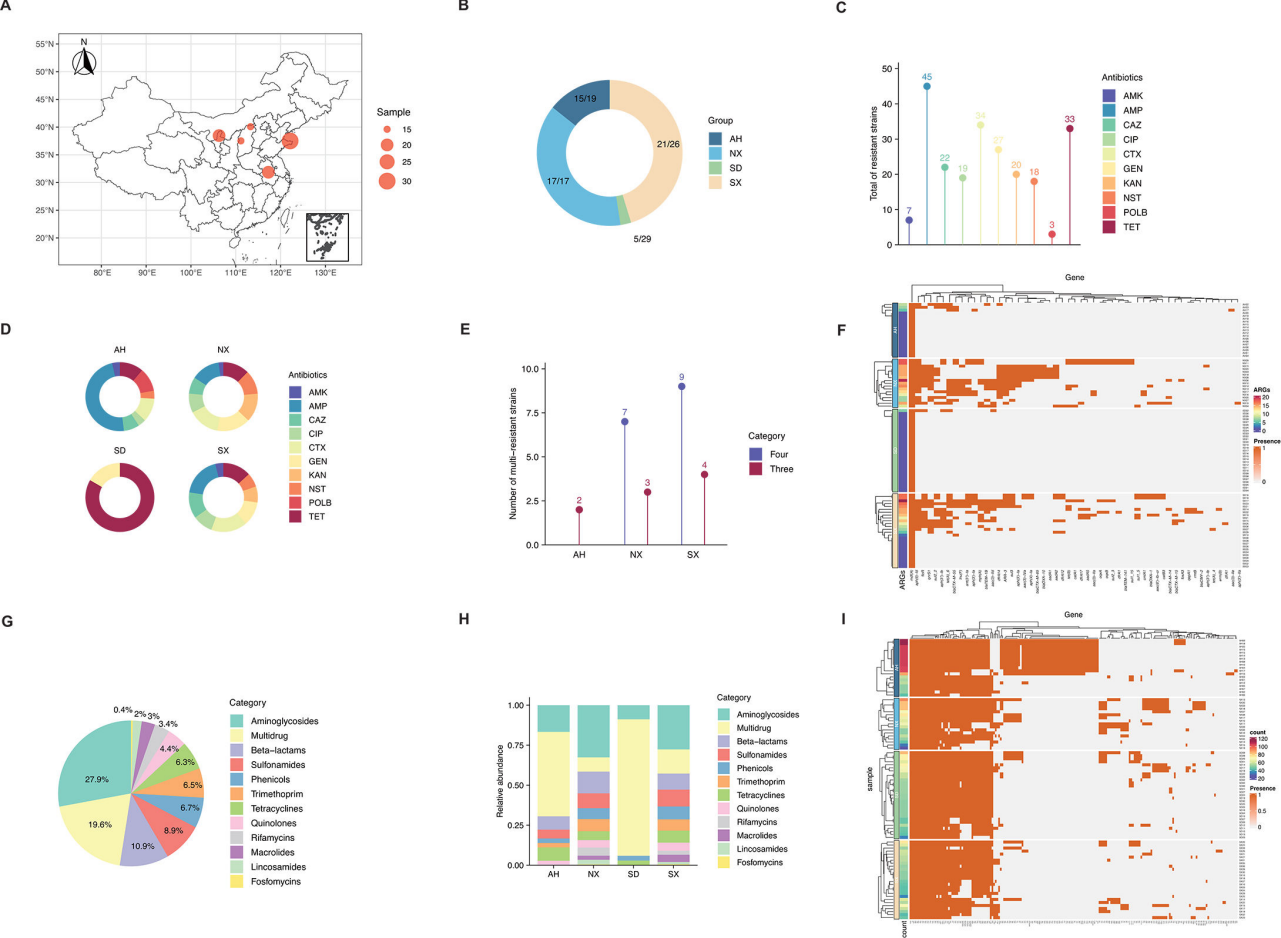
Geographical distribution, antimicrobial resistance profiles, and genomic characteristics of bovine *E. coli* isolates. The map was created using R (packages ggplot2, ggspatial, and cowplot). (**A**) Geographic distribution of sampling sites across four provinces in China; the size of red circles represents the number of samples collected in each province. (**B**) Number of resistant isolates identified in each province; colors denote different provinces. (**C**) Number of resistant isolates against ten tested antibiotics; colors indicate different antibiotics, and numbers above the bars represent the counts of resistant isolates. (**D**) Distribution of antibiotic-resistant isolates across provinces for each antibiotic. (**E**) Distribution of multidrug-resistant (MDR) isolates among provinces; dark red indicates triple-drug resistance, and dark purple indicates quadruple-drug resistance. (**F**) Heatmap of antibiotic resistance gene (ARG) annotations; the purple-to-red gradient represents the number of resistant isolates, while orange and gray denote presence and absence of specific ARGs, respectively. (**G**) Overall distribution of ARG categories among all isolates; colors represent distinct antibiotic classes. (**H**) Distribution of ARG categories across provinces; color scheme consistent with panel **G**. (**I**) Heatmap of virulence factor gene (VFG) annotations; color scheme consistent with panel **F**.

A total of 91 *E. coli* strains were isolated from 100 fecal samples collected across four provinces, including Anhui (AH), Ningxia (NX), Shandong (SD), and Shanxi (SX) (Fig. 1A). All isolates were successfully identified using MALDI-TOF mass spectrometry and further confirmed by average nucleotide identity (ANI) based on whole genome sequencing (WGS) data.

Subsequently, ten antibiotics across five antibiotic classes were included and employed to determine AMR of isolates. There were found 58 resistant strains in 91 isolates that were resistant to at least one antibiotic (Fig. 1B). Isolates from the province of NX had the highest resistance prevalence in contrast with other provinces, while SD had the lowest (Fig. 1B). Resistance to ampicillin (AMP) was most frequently observed, followed by cefotaxime (CTX), tetracyclines (TET), gentamicin (GEN), ceftazidime (CAZ), kanamycin (KAN), ciprofloxacin (CIP), neomycin sulfate (NST), amikacin (AMK), and polymyxin B (POLB) (Fig. 1C). Notably, isolates resistant to AMP predominated in both AH and SX, while resistance to GEN and TET was more common in NX and SD (Fig. 1D). Among all provinces, SD had the fewest resistant isolates (*n* = 5), with resistance observed only to TET and GEN (Fig. 1D).

Additionally, we further analyzed the distribution of MDR isolates across the four provinces. Twenty-one *E. coli* strains were identified as MDR, all originating from AH, NX, and SX (Fig. 1E). Notably, 16 of these isolates, from SX and NX, exhibited resistance to four classes of antibiotics (Fig. 1E).

### Annotation of ARGs, VFGs, and plasmids

Genome assembly quality has been assessed using QUAST v5.2.0, and the key statistics, including genome size, number of contigs, N50, and GC content, have been summarized in a supplemental table (Table S1). To further investigate the AMR and virulence characteristics of bovine *E. coli*, ARGs, VFGs, and plasmids were annotated based on WGS data. Altogether, 53 distinct ARGs were identified, with a total of 505 gene occurrences detected across the four provinces (Fig. 1F). Of these, *mdf*(*A*), *aph*(6)*-Id*, *tet*(*A*), and *floR* were among the most frequently detected ARGs, each with more than 20 occurrences (Table S2). NX and SX had the highest numbers of detected ARGs, suggesting these regions bear a greater AMR burden (Fig. 1F). In addition, we found these ARGs were dominated by aminoglycosides, followed by multidrug, beta-lactams, sulfonamides, phenicols, and other categories (Fig. 1G). Genes related to multidrug were dominant in AH and SD, while aminoglycosides were dominant in NX and SX (Fig. 1H). We further analyzed ARGs at the class level across the four provinces; however, no significant variation was found (Fig. S1A).

**Fig 2:**
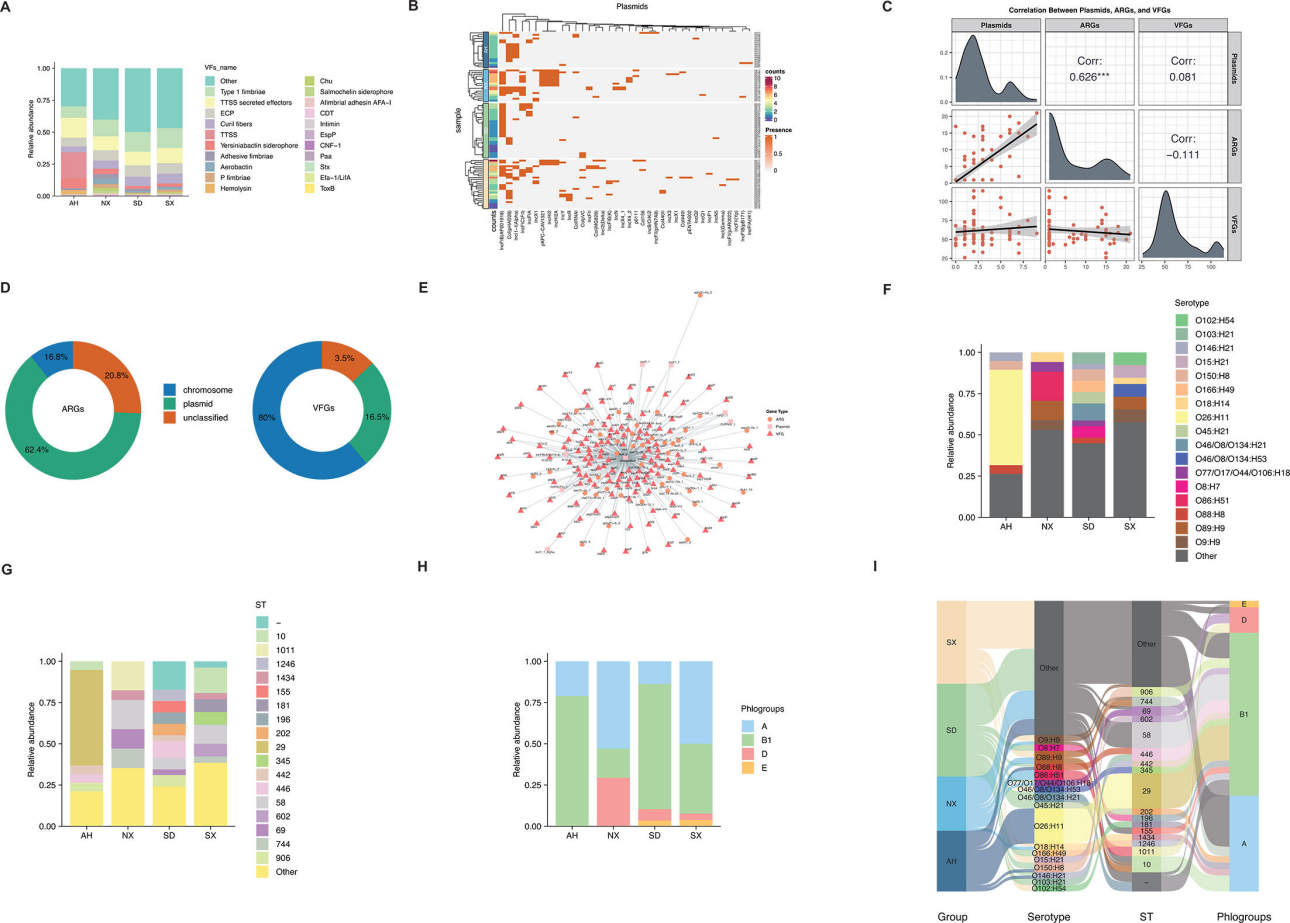
Distribution of virulence factors, plasmid characteristics, and population structure of bovine *E. coli* isolates. (**A**) Distribution of virulence factor (VF) categories across four provinces; colors represent different VF types. (**B**) Heatmap of plasmid replicon annotations; the purple-to-red gradient indicates the number of plasmids per isolate, while orange and gray denote presence and absence, respectively. (**C**) Correlation analysis among plasmids, antibiotic resistance genes (ARGs), and virulence factor genes (VFGs); ***, *P* < 0.01. (**D**) Genomic localization of ARGs and VFGs within isolates; blue indicates chromosomal, green plasmid, and orange unclassified positions. (**E**) Co-occurrence network of plasmids, ARGs, and VFGs; orange circles denote ARGs, pink squares plasmids, and red triangles VFGs. (**F**) Distribution of *E. coli* serotypes across provinces; colors represent distinct serotypes. (**G**) Distribution of sequence types (STs) across provinces; colors represent distinct STs. (**H**) Distribution of phylogroups among isolates from four provinces; colors represent different phylogroups. (**I**) Sankey diagram illustrating relationships between provinces (left), serotypes, STs, and phylogroups (right); flow width corresponds to isolate counts.

Across the four provinces, 196 types of VFGs were detected, totaling 5,632 gene occurrences, with AH exhibiting the greatest virulence gene burden (Fig. 1I). The most frequently detected VFGs were primarily related to adherence (*fim* operon genes, *csg* family, and *ecp* cluster) and effector delivery systems (*espX1*, *espX4*, *espX5*, *espR1*, and *espL1*), suggesting strong virulence potential among the isolates (Fig. 1I). Following this, virulence genes were classified according to their designated names and associated virulence categories (Fig. S1B). Adherence-related systems were the most dominant, followed by effector delivery systems, nutritional/metabolic factors, exotoxins, and others (Fig. S1B). Type 1 fimbriae were the most prevalent virulence factors, followed by type III secretion system (TTSS)*-*secreted effectors*, Escherichia coli* common pilus (ECP), curli fibers, general TTSS components, and others (Fig. S1C). Notably, the Yersiniabactin siderophore system was particularly prevalent in AH, while similar prevalence patterns were observed across the other provinces (Fig. 2A).

Based on WGS data, plasmid replicon types were identified to explore their distribution. A total of 37 distinct plasmid replicon types were identified across all isolates. Among them, IncFIB was the most prevalent, followed by Col(pHAD28), IncI1-I, IncFIC(FII), IncHI2A, and others (Fig. 2B; Table S2). NX exhibited the highest number of plasmids (83), with SX (73), SD (39), and AH (37) reporting progressively fewer detections (Fig. 2B). Moreover, the most frequently identified plasmid in NX, SD, and SX was IncFIB, whereas Col(pHAD28) was the top type in AH (Fig. S1D).

### Correlation and network analysis of plasmid-associated resistance and virulence genes

Correlation analysis was employed to reveal the relationship of plasmids with ARGs and VFGs based on the annotation results. We found a strong positive association between plasmids and ARGs (*r* = 0.626, *P *< 0.001), but no significant correlations between plasmids and VFGs (Fig. 2C). Significant correlations between plasmids and ARGs were specifically detected in NX (*r* = 0.558, *P* < 0.05), while strong plasmid-VFG associations were observed in AH (*r* = 0.551, *P *< 0.05), with no evident trends in the other provinces (Fig. S1E through H).

Following the correlation analysis, we further employed PlasFlow to predict whether ARGs and VFGs were located on plasmids or chromosomes. The results indicated that the majority of VFGs were located on chromosome-derived contigs, whereas ARGs showed a strong tendency to be plasmid-associated, suggesting differing mechanisms of gene dissemination (Fig. 2D; Fig. S1I and J). Therein, the opposite trend was observed in SD due to the limited resistant strains (Fig. S1J).

To better understand the genomic localization of resistance and virulence genes, we integrated the annotation results of ARGs, VFGs, and plasmids to identify their presence on specific plasmid types. Due to the limitations of short-read sequencing, only 19 strains allowed confident localization of these genes on well-defined plasmids (Table S1). In these strains, *bla*_CTX-M-55_ was detected on IncI1_1 plasmid. IncX1, particularly in NX07 and SX29, commonly carried *aph*(6)-*Id* and *tet*(A), while IncQ1 (NX17) and ColRNAI (SX14) co-carried multiple resistance genes, suggesting roles in multidrug resistance (Table S2; Fig. 2E). For virulence genes, IncFIB plasmids in SD08 and SX20 carried both *iuc* and *f17d* operons, whereas SD01 harbored *iuc* and *cdt* clusters on the same plasmid. AH12 contained a complete *hly* operon on IncFIB, and *fae* operons were found on IncFIC(FII) in SD12 and SD14. Many resistance and virulence genes were plasmid-borne, with network analysis suggesting that some untyped plasmids may serve as hubs (Fig. 2E). These findings underscore the need for combined long- and short-read sequencing to improve plasmid resolution and contextual analysis.

### Molecular typing of *E. coli* isolates

A total of 59 O:H serotypes were identified, with O26:H11 (*n* = 11) being the most prevalent, followed by O89:H9 (*n* = 4) and several serotypes with three isolates each (O150:H8, O86:H51, O9:H9) (Table S2). Serotype distribution exhibited distinct regional characteristics, with O26:H11 detected only in AH, while O86:H51 and O89:H9 were mainly found in NX (Fig. 2F). While certain serotypes like O88:H8 and O88:H9 appeared across regions, others displayed region-specific distribution (Fig. 2F). Several serotypes shared overlapping O antigens or lacked identifiable O types (“–:H”), suggesting potential cross-reactivity, novel serotypes, or unresolved antigenic assignments (Table S2). The overall diversity reflects complex local transmission dynamics and genetic variation among isolates.

ST types exhibited notable regional patterns and diversity, with a total of 45 distinct types identified. Therein, ST29 was predominant and exclusively detected in AH, while ST58 was identified across all four regions (Fig. 2G). Several STs, such as ST446 and ST906, were shared between AH and SD, whereas others, like ST1011 and ST744, were limited to NX and SX (Fig. 2G). A few strains remained untyped, indicating the presence of potentially novel or unclassified STs.

Ninety-one isolates were classified into four phylogroups, with B1 (*n *= 51) and A (*n *= 30) being the most prevalent (Fig. 2H; Table S2). B1 was dominant in AH and SD, while A was more frequent in SX and NX (Fig. 2H). Phylogroups D and E were infrequent, primarily identified in NX, SD, and SX (Fig. 2H). These patterns indicate potential regional variations; however, the sparse sampling density necessitates careful interpretation.

Furthermore, the Sankey diagram revealed strong regional links between ST types, phylogroups, and serotypes. AH was dominated by ST29 and phylogroup B1, mainly with serotype O26:H11 (Fig. 2I). NX showed diverse STs and phylogroups A and D, with unique serotypes like O86:H51 and O89:H9 (Fig. 2I). SD mainly had phylogroup B1 and ST446, associated with O88:H8 and O146:H21 (Fig. 2I). SX exhibited varied STs and mostly phylogroups A and B1, sharing some serotypes with other regions (Fig. 2I). These results highlighted clear geographic patterns in genetic diversity.

### Phylogenetic analysis of *E. coli* isolates

**Fig 3:**
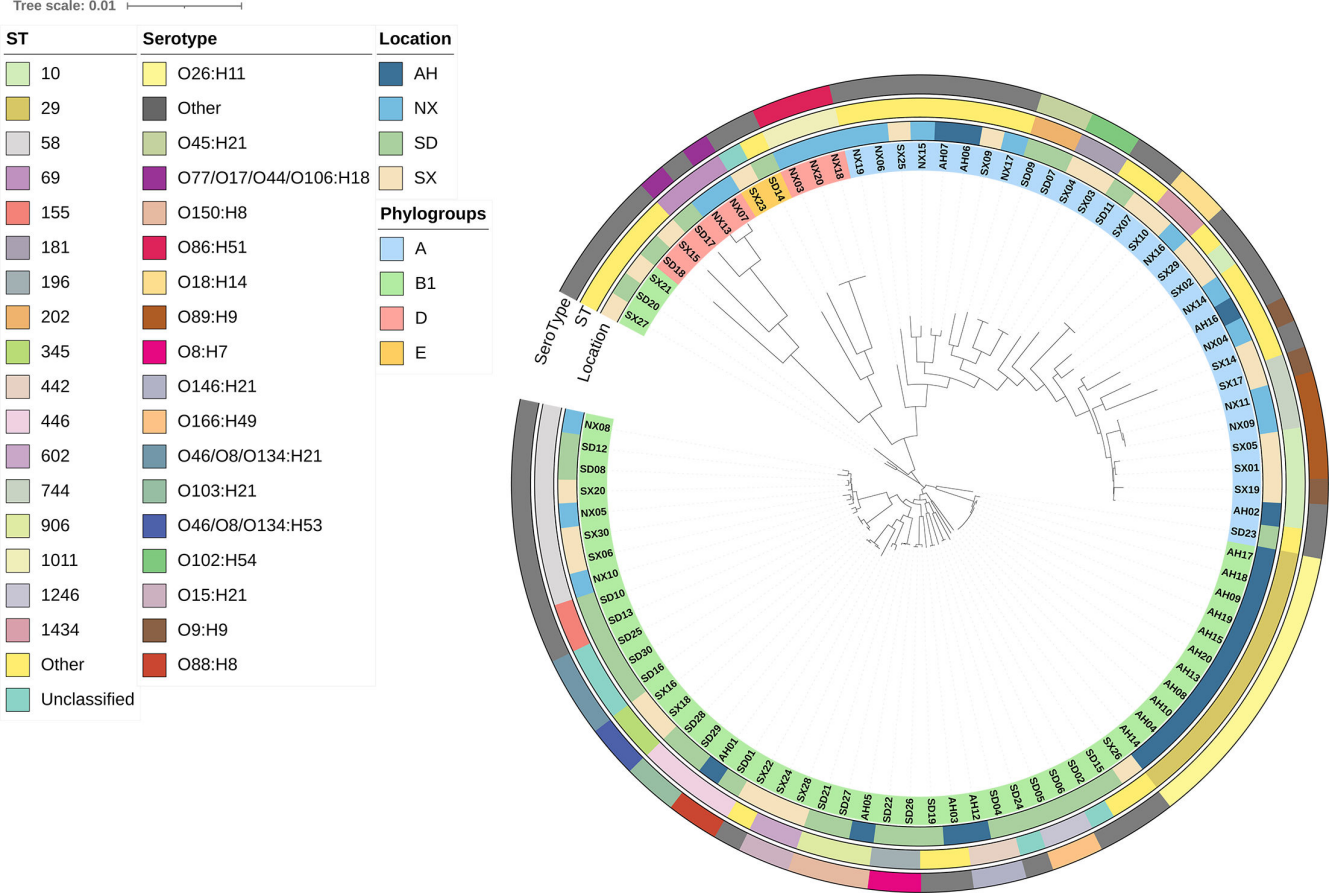
Phylogenetic analysis and genomic feature distribution of 91 bovine *E. coli* isolates. Circular phylogenetic tree based on core genome alignment. From inside out: phylogroups, geographic origin, sequence types (STs), and serotypes.

We constructed a phylogenetic tree based on core genomes to elucidate the evolutionary relationships among the 91 isolates. The results showed that strains belonging to the same phylogroup clustered together regardless of their provincial origin, indicating a phylogroup-driven clustering pattern (Fig. 3). The only exception was strain SD23, which, although classified as phylogroup A, grouped with B1 strains. Moreover, distinct clonal structures were observed within phylogroups, with ST10 dominating phylogroup A, ST29 and ST58 being most frequent in phylogroup B1, and ST1011 representing the major lineage within phylogroup D (Fig. 2I and 3).

Sequence types such as ST29, ST446, ST906, and ST1011 were linked to specific serotypes, including O26:H11, O150:H8, and O86:H51 (Fig. 3). In phylogroup B1, strains often clustered with similar serotypes, reflecting clonal and phylogenetic relationships. The phylogenetic clustering of strains from different provinces suggests potential intra-provincial transmission, emphasizing the need for sustained genomic monitoring to track and manage the spread of these lineages.

## DISCUSSION

In this study, *E. coli* was isolated from 91% of bovine fecal samples with high rates collected from four provinces in China, which is consistent with the fact that *E. coli* is a common commensal organism in the intestinal tract of animals (23). Importantly, antimicrobial susceptibility testing revealed that 63.7% (58/91) of the isolates were resistant to at least one antibiotic, with MDR strains accounting for 23.1% (21/91). These findings underscore a substantial AMR burden in bovine *E. coli*, aligning with global concerns about the overuse of antibiotics in animal husbandry (7). Resistance to AMP, TET, and GEN was most common, in agreement with the latest reports on *E. coli* from livestock in Nairobi, Kenya (24). Isolates from NX exhibited the highest resistance levels, suggesting a substantial AMR burden, whereas those from SD showed the lowest, possibly reflecting more prudent antibiotic use or effective farm management (25).

To further explore the genetic basis of AMR and virulence in bovine *E. coli*, we analyzed the distribution of antibiotic resistance genes (ARGs) and VFGs across the isolates. Among the 53 distinct ARGs identified, *mdf*(A), *aph*(6)-*Id*, *tet*(A), and *floR* were the most prevalent. These genes have been widely recognized as major contributors to AMR in livestock-associated *E. coli*, in line with prior reports from China (26). Isolates from NX and SX harbored the highest numbers of ARGs, corresponding with the observed resistance phenotypes, but multidrug resistance genes were common in AH and SD. The findings indicated that disparities in resistance patterns might be caused by regional variations in farming practices, livestock density, and antibiotic use, as well as comparatively weak biosecurity measures (27). In terms of virulence genes, 196 distinct types of VFGs were detected, with AH displaying the highest virulence burden. Overall, adhesion-associated genes (*fim*, *csg*, and *ecp*) and type III secretion system effectors (*espX* and *espL*) were particularly prevalent, which is similar to the findings from dairy farm-derived *E. coli* in Gansu province (28). Furthermore, AH isolates have much higher levels of yersiniabactin. This siderophore has been linked to increased iron acquisition and virulence, and prior research has revealed that yersiniabactin may promote intestinal inflammation by activating host immunological pathways, such as NLRP3 inflammasome signaling (29). In addition, we discovered that a few isolates from AH were members of the ST29 and serotype of O26:H11, which has been linked to human infections with Shiga toxin-producing *E. coli* (STEC) (30). This raises questions regarding the zoonotic significance and possible pathogenicity of yersiniabactin-positive strains in cattle. These findings underscore the public and animal health risks posed by *E. coli* strains that concurrently harbor multiple resistance and virulence determinants. Ongoing surveillance of STEC in cattle is essential for ensuring food safety and public health, given that bovines are acknowledged reservoirs of human illnesses, with multiple STEC outbreaks associated with beef and dairy production reported in China (31, 32).

Given the critical role of plasmids in mediating HGT, we investigated the associations between plasmid replicons and the distribution of ARGs and VFGs (11). In our study, 37 plasmid replicon types were identified, with IncFIB being the most prevalent, particularly in NX, SX, and SD, which is consistent with previous reports of its role in disseminating resistance genes among livestock-associated *E. coli* (33). Correlation analysis revealed a significant association between plasmids and ARGs, especially in NX, indicating region-specific plasmid-mediated resistance transmission. Although most VFGs were chromosomally encoded, a notable plasmid-VFG association in AH isolates suggests the occasional mobilization of virulence determinants. Additionally, certain plasmids (IncI1, IncQ1, and IncF-type) co-harbored multiple ARGs such as *bla*_CTX-M-55_, *aph*(6)-*Id*, and *tet*(A), and in some cases, virulence operons like *iuc*, *cdt*, and *hly*, underscoring the important role of plasmids in disseminating both resistance and virulence genes (34). While plasmids are central to resistance and virulence transmission, numerous gene-linked plasmids remain unclassified owing to current sequencing limitations, underscoring the value of integrating long- and short-read sequencing for improved resolution in the future.

The predominance of *E. coli* serotype O26:H11, particularly linked to ST29 exclusively in AH, is noteworthy due to its recognition as a highly virulent Shiga toxin-producing EHEC clone implicated in severe infections in both humans and cattle globally (19). The geographic confinement of O26:H11 in AH highlights a region-specific epidemiological concern, potentially indicative of zoonotic spillover risk. Notably, previous studies have demonstrated that ST29/O26:H11 lineages are genetically diverse, with several clades associated with Shiga toxin production and enhanced virulence in both experimental models and human infections (26, 35). In contrast, ST58 was detected in all regions and, despite lacking ColV plasmids, carried virulence factors like Yersiniabactin, indicating potential pathogenicity in animal hosts (36). This suggests livestock may serve as reservoirs for potentially pathogenic *E. coli*. Serotype diversity was evident with O86:H51 and O89:H9 predominating in NX, and their unclear pathogenic roles warrant further investigation in animal reservoirs. Moreover, regional recurrence of ST446 and ST906, often linked with O88:H8 and O150:H8 in AH and SD, may indicate localized clonal persistence, consistent with established *E. coli* population dynamics (17). Collectively, these findings reveal region-specific lineage patterns and broader genetic exchange, underscoring the value of genomic surveillance in monitoring bovine *E. coli* diversity and its potential implications for animal and public health.

The core genome phylogeny revealed clear clustering patterns primarily driven by phylogroup affiliation rather than geographic origin. Notably, strains from different provinces clustered closely within the same phylogenetic groups, indicating that identical or closely related lineages are present across multiple regions. This pattern suggests potential inter-provincial transmission or common sources of these *E. coli* clones in cattle, a trend also reported in ovine isolates (37). Notably, ST58, detected across multiple provinces, is a globally disseminated lineage often associated with multidrug resistance, while O26:H11/ST29 and region-specific ST1011 isolates showed localized clustering, and plasmid-mediated resistance genes such as *bl*a_CTX-M-55_ and *tet*(A) were enriched in certain clonal backgrounds, highlighting the interplay between clonality and resistome composition (36). In addition, strains with the same serotype (such as O150:H8 or O86:H51) often clustered together, even when isolated from different provinces and belonging to different STs (including ST446, ST906, and ST1011), reflecting the complex relationship between antigenic diversity and genetic background. These observations highlight the need for ongoing genomic surveillance to track clonal spread and elucidate the transmission dynamics of potentially zoonotic or pathogenic *E. coli* strains. One drawback of our investigation is the lack of worldwide *E. coli* isolates from diarrheic cattle for comparison analysis; thus, the extent to which Chinese strains differ in gene content or phylogenetic position from international equivalents remains to be determined.

### Conclusions

This study demonstrated a high prevalence and substantial genetic diversity of antimicrobial-resistant *E. coli* in bovine fecal samples from four Chinese provinces. A total of 91 isolates were recovered, with 23.1% exhibiting MDR, particularly against ampicillin, tetracycline, and gentamicin. Whole-genome sequencing identified diverse ARGs, many of which were plasmid-borne, especially on IncI1, IncX1, and IncFIB types, suggesting HGT as a key mechanism for resistance spread. Virulence genes associated with adhesion and secretion systems were primarily chromosomally encoded, with isolates from AH province harboring the highest virulence gene burden. Molecular typing uncovered region-specific STs and serotypes, while phylogenetic analyses indicated both clonal expansion and inter-provincial transmission events. Collectively, our findings highlight the genomic complexity and regional variation of cattle-associated *E. coli* in China, underscore the zoonotic potential of MDR strains, and reinforce the need for genomic surveillance and prudent antibiotic use within an integrated One Health framework.

## MATERIALS AND METHODS

### Sample information and study context

China is the largest beef producer in Asia and the third largest in the world, and its cattle industry is growing quickly and includes both industrial and native breeds (38). Cattle are mostly found in the northern and western parts of China, like Inner Mongolia, Heilongjiang, NX, and Shanxi, where beef and dairy farming are most common (38). In general, cattle farming can be done in a lot of different ways. Some families run small herds with limited biosecurity, while others run large commercial feedlots that use more intensive management methods. In this study, a total of 100 fresh fecal samples were randomly collected from diarrheic cattle on several scale farms in four provinces of China, namely AH, NX, SD, and SX. Among these, 20 samples were collected from each of AH and NX, while 30 samples were collected from SD and SX.

### Strain collections and identification

All samples were promptly transported to the laboratory on ice after collection. Each sample was pre-enriched in 5 mL of buffered peptone water at 37°C for 4 h. The pre-enriched cultures were then streaked onto MacConkey agar plates for preliminary isolation of *E. coli*. Colonies with typical *E. coli* morphology were subsequently cultured onto Eosin Methylene Blue agar for further confirmation and purification. The selected strains were purified through two successive passages on LB agar and then stored in 25% glycerol at −80°C for subsequent analyzes. Species-level identification of the bacterial isolates was conducted using matrix-assisted laser desorption ionization time-of-flight mass spectrometry (MALDI-TOF MS).

### Antimicrobial susceptibility testing

All isolates were streaked onto LB agar and incubated overnight to ensure optimal growth prior to antimicrobial susceptibility testing. Susceptibility profiles were determined using the Kirby-Bauer disk diffusion method against a panel of ten antibiotics, representing five key antimicrobial classes: aminoglycosides (amikacin, gentamicin, kanamycin, and neomycin sulfate), β-lactams (cefotaxime, ceftazidime, and ampicillin), quinolones (ciprofloxacin), tetracyclines (tetracycline), and polypeptides (polymyxin B). The abbreviations AMK, GEN, KAN, NST, CTX, CAZ, AMP, CIP, TET, and POLB are used throughout to denote these antibiotics. *E. coli* ATCC 25922 was used as the quality control strain. Antibiotic resistance was assessed in accordance with Clinical and Laboratory Standards Institute guidelines (39).

### Whole-genome sequencing and assembly

The TIANamp Bacteria DNA Kit (Tiangen, Beijing, China) was employed to extract genomic DNA from the isolates, following the supplier’s recommended protocol.

DNA concentration and purity were measured using a NanoDrop One spectrophotometer (Thermo Fisher Scientific, USA). Genomic libraries were constructed and subjected to 150 bp paired-end sequencing on the DNBSEQ-T7 platform (BGI Genomics Co., Ltd., Shenzhen, China). Adapter sequences and low-quality bases were removed using fastp v0.23.4 (40), and high-quality reads were subsequently assembled into draft genomes using SPAdes v3.15.5 (41).

### Bioinformatic analysis

The quality of genome assemblies was assessed using QUAST v5.2.0 (42). To reconfirm species identity, ANI was calculated using FastANI v1.34 (43), comparing each genome to the reference *E. coli* strain GCF_000005845.2 from the NCBI RefSeq database. Genome annotation was performed with Prokka v1.14.6 (44), followed by core genome alignment using Roary v3.13.0 (45). A maximum-likelihood phylogenetic tree was constructed based on the core genome using FastTree v2.1 (https://morgannprice.github.io/fasttree/), and further visualized using the Interactive Tree of Life platform (46). Antibiotic resistance genes (ARGs), VFGs, and plasmid replicons were identified using Abricate v1.0.1 (https://github.com/tseemann/abricate) against the ResFinder, Virulence Factor databse (VFDB), and PlasmidFinder databases (47–49). All gene and replicon names are presented as reported by the corresponding databases, including suffix identifiers to maintain consistency with standardized nomenclature. *In silico* multilocus sequence typing was carried out with mlst v2.23.0 (https://github.com/tseemann/mlst) (50), while phylogroups were determined using ClermonTyping (16). Serotypes were predicted using ECTyper v1.0.0 (51). All data analyses and visualizations were performed using R v4.4.0 and associated packages.

### Integrative analysis of ARGs and VFGs on plasmids

To investigate the associations between plasmids and resistance or virulence genes, we first performed a correlation analysis based on annotation results from PlasmidFinder, ResFinder, and VFDB. To further determine whether ARGs and VFGs were located on chromosomes or plasmids, PlasFlow was used to predict the origin of genomic contigs (52). The presence of plasmid-associated ARGs and VFGs was subsequently validated using results from PlasmidFinder. Finally, an association network was constructed using R to visualize the co-occurrence patterns between plasmids and resistance or virulence genes.

### Statistical analysis

The Wilcoxon exact test was used to compare the distributions of different classes of ARGs and VFGs across the four provinces because the data did not follow a normal distribution. Pearson’s correlation coefficient was employed to assess the relationships between plasmid types and the existence of resistance and virulence genes. Using the Benjamini–Hochberg method, the *P*-values were adjusted for multiple comparisons. Values of *P *< 0.05 were seen as statistically significant. All statistical analyses and data visualizations were executed using R version 4.4.0.

## Data Availability

The data that support the findings of this study have been deposited into CNGB Sequence Archive (CNSA) (53) of China National GeneBank DataBase (CNGBdb) (54) with accession number CNP0007098.

## SUPPLEMENTAL MATERIAL

Fig. S1 spectrum.03256-25-s0001.pdf Comparative distribution and genomic localization of antibiotic resistance genes (ARGs), virulence factor genes (VFGs), and plasmids in bovine *E. coli* isolates from four provinces.

Supplemental legends spectrum.03256-25-s0002.docx Legends for Fig. S1 and Tables S1 and S2.

Table S1 spectrum.03256-25-s0003.xls Genome assembly quality and antibiotic resistance gene (ARG) annotations of bovine *E. coli* isolates.

Table S2 spectrum.03256-25-s0004.xlsx Phenotypic resistance profiles and genomic characteristics of bovine *E. coli* isolates.

ASM does not own the copyrights to Supplemental Material that may be linked to, or accessed through, an article. The authors have granted ASM a non-exclusive, world-wide license to publish the Supplemental Material files. Please contact the corresponding author directly for reuse.
