# Supplementary figures and images for: Genomic epidemiology and plasmid characterization of antimicrobial resistance and virulence in cattle Escherichia coli from China
Source: Microbiol Spectr. 2025 Dec 4;14(1):e03256-25. doi: 10.1128/spectrum.03256-25 (PMC12772300; doi:10.1128/spectrum.03256-25)

A

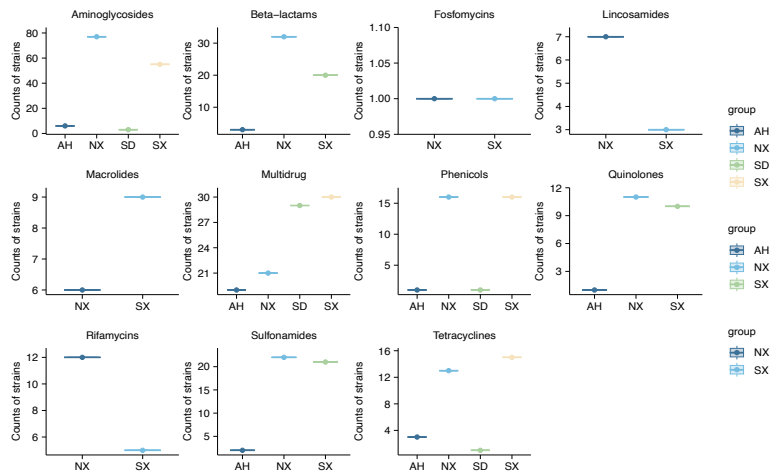

B

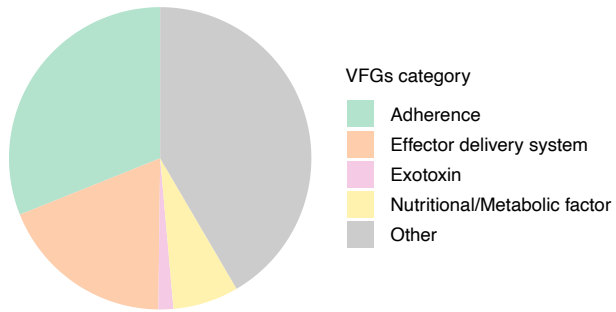

C

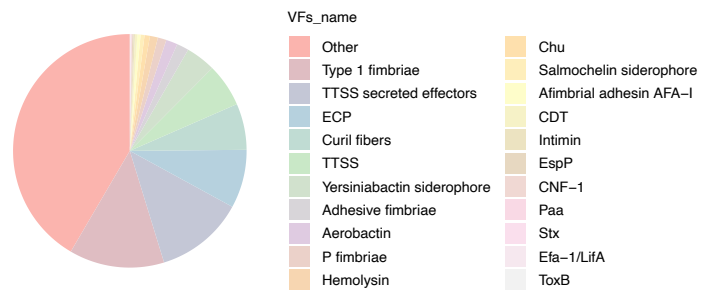

D

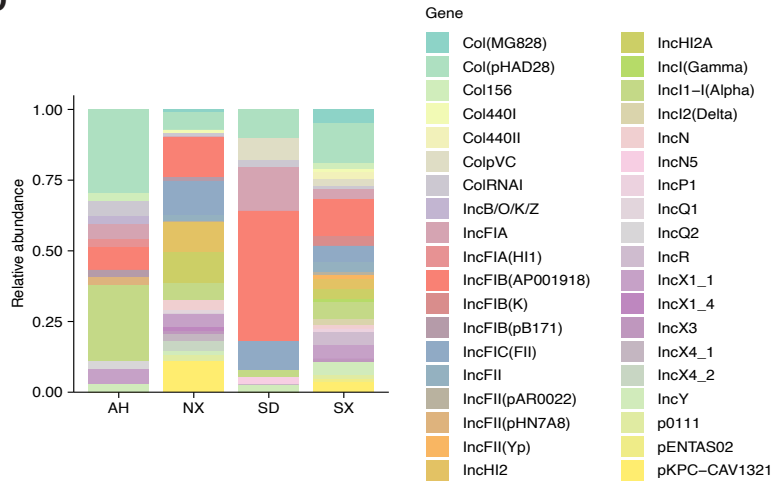

E

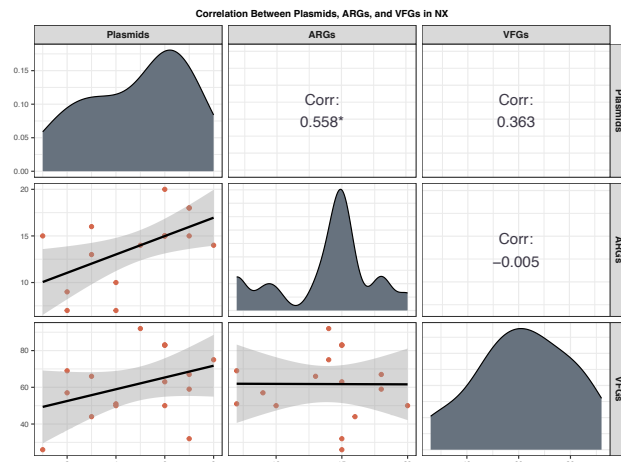

F

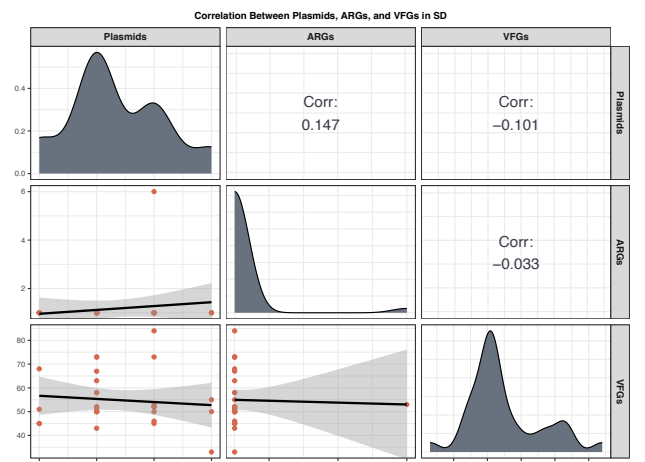

G

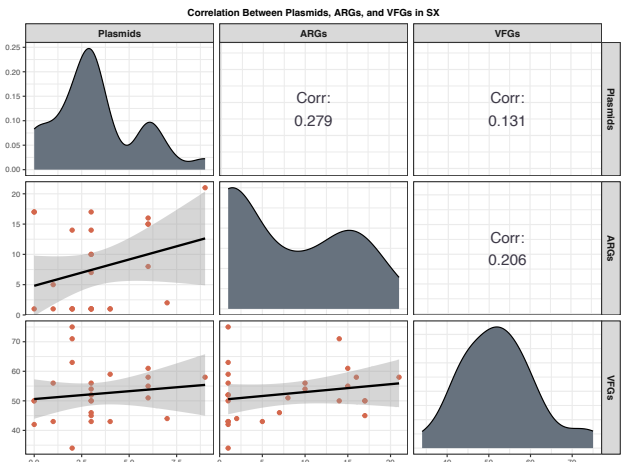

H

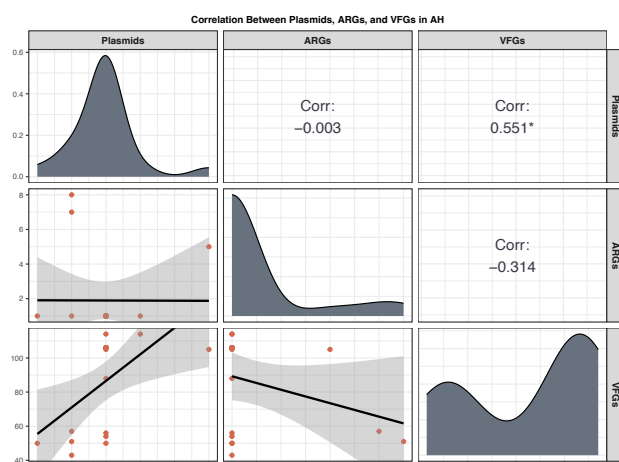

I

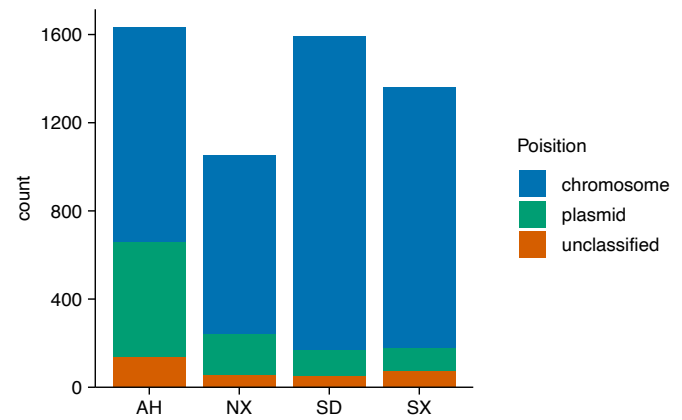

J

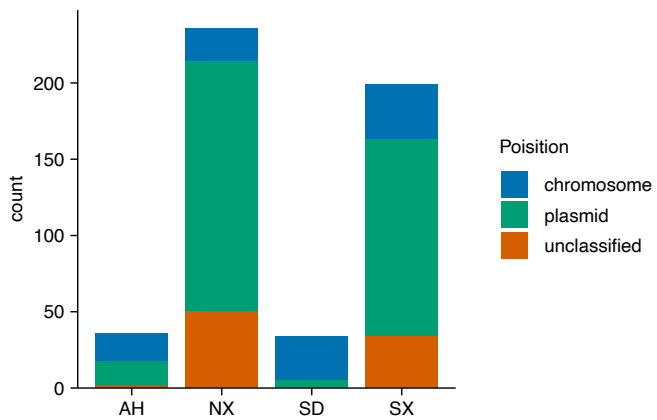

Supplement: Fig. S1 — Comparative distribution and genomic localization of antibiotic resistance genes (ARGs), virulence factor genes (VFGs), and plasmids in bovine E. coli isolates from four provinces. [file spectrum.03256-25-s0001.pdf]
