# Supplementary material for: Genomic epidemiology and plasmid characterization of antimicrobial resistance and virulence in cattle Escherichia coli from China
Source: Microbiol Spectr. 2025 Dec 4;14(1):e03256-25. doi: 10.1128/spectrum.03256-25 (PMC12772300; doi:10.1128/spectrum.03256-25)
Supplement: Supplemental legends — Legends for Fig. S1 and Tables S1 and S2. [file spectrum.03256-25-s0002.docx]

### SUPPLEMENTAL MATERIAL

Fig. S1. Comparative distribution and genomic localization of antibiotic resistance genes (ARGs), virulence factor genes (VFGs), and plasmids in bovine *E. coli* isolates from four provinces.

(A) Differential abundance analysis of ARG categories across four provinces, grouped by antibiotic class.

(B) Overall distribution of virulence systems among all isolates; different colors represent distinct virulence systems.

(C) Overall distribution of virulence factors; colors denote different virulence factor types.

(D) Distribution of plasmid replicon types across provinces; colors represent different plasmid types.

(E–H) Correlation analyses between plasmids, ARGs, and VFGs in Ningxia (E), Shandong (F), Shanxi (G), and Anhui (H); *, P < 0.05.

(I) Distribution of genomic locations of ARGs across provinces; blue indicates chromosomal, green plasmid, and orange unclassified positions.

(J) Distribution of genomic locations of VFGs across provinces; color scheme consistent with panel (I).

Table S1. Genome assembly quality and antibiotic resistance gene (ARG) annotations of bovine *E. coli* isolates.

This supplementary table comprises two worksheets providing genome assembly metrics and antibiotic resistance gene annotations.

Sheet 1-Quast_res: Genome assembly quality statistics generated using QUAST v5.2.0, including total genome length, GC content, number of contigs, and N50 values for each isolate.

Sheet 2-Gene: Annotated antibiotic resistance genes (ARGs) identified in all isolates, along with their corresponding antibiotic classes and resistance categories.

Table S2. Phenotypic resistance profiles and genomic characteristics of bovine *E. coli* isolates.

This supplementary table contains eight worksheets summarizing phenotypic and genomic features associated with antimicrobial resistance and virulence.

Sheet 1-Phenotype: Antimicrobial susceptibility results showing resistance or susceptibility of each isolate to ten antibiotics.

Sheet 2-ARGs: Summary of annotated ARGs, presenting the number of genes detected.

Sheet 3-VFGs: Summary of annotated virulence factor genes (VFGs), categorized by virulence system and virulence factor type.

Sheet 4-Plasmid: Distribution and number of plasmid replicons detected per isolate.

Sheet 5-Co-exit: Co-localization patterns of ARGs and VFGs based on PlasFlow prediction results, indicating whether genes are located on plasmids, chromosomes, or unclassified regions.

Sheet 6-Serotype: Identified serotypes and their frequencies across all isolates.

Sheet 7-ST: Sequence types (STs) assigned to isolates based on multilocus sequence typing (MLST) analysis.

Sheet 8-Phylogroups: Classification and distribution of phylogroups among all *E. coli* isolates.
